# Comprehensive prognostic report of the Japanese Breast Cancer Society Registry in 2005

**DOI:** 10.1007/s12282-015-0645-4

**Published:** 2015-10-13

**Authors:** Keisei Anan, Naohito Fukui, Takayuki Kinoshita, Takayuki Iwamoto, Naoki Niikura, Masaaki Kawai, Naoki Hayashi, Kouichiro Tsugawa, Kenjiro Aogi, Takanori Ishida, Hideji Masuoka, Sinobu Masuda, Kotaro Iijima, Seigo Nakamura, Yutaka Tokuda

**Affiliations:** Department of Surgery, Kitakyushu Municipal Medical Center, Kitakyushu, Japan; The Japan Clinical Research Support Unit and the Public Health Research Foundation, Tokyo, Japan; Department of Breast Surgery, National Cancer Center Hospital, Tokyo, Japan; Department of Breast and Endocrine Surgery, Okayama University Hospital, Okayama, Japan; Department of Breast and Endocrine Surgery, Tokai University School of Medicine, 143 Shimokasuya, Isehara, Kanagawa 259-1193 Japan; Department of Breast Surgery, Miyagi Cancer Center, Natori, Japan; Department of Breast Surgery, St. Luke’s International Hospital, Tokyo, Japan; Division of Breast and Endocrine Surgery, Department of Surgery, St. Marianna University School of Medicine, Kawasaki, Japan; Department of Breast Surgery, Shikoku Cancer Center, Matsuyama, Japan; Department of Surgical Oncology, Graduate School of Medicine, Tohoku University, Sendai, Japan; Sapporo-kotoni Breast Clinic, Sapporo, Japan; Department of Pathology, Nihon University School of Medicine, Tokyo, Japan; Department of Breast Oncology, Cancer Institute Hospital, Tokyo, Japan; Division of Breast Surgical Oncology, Department of Surgery, Showa University, Tokyo, Japan

**Keywords:** Breast cancer, Prognosis, Report, Japan, Registry, 2005, The Japanese Breast Cancer Society

## Preface

A population-based cancer registry has been used for the planning and evaluation of cancer control activities based on administration and the care of individual cancer patients by those in the medical profession. The Japanese Breast Cancer Society (JBCS) registry was started in 1975. In 2004, the registry system was moved to a new system using web registration with the cooperation of the Non-Profit Organization Japan Clinical Research Support Unit and Public Health Research Foundation (Tokyo, Japan). Comprehensive individual patient data were recorded according to the Unio Internationalis Contra Cancrum (UICC) TNM classification [1] and the World Health Organization histological classification [2]. The details are described elsewhere [3]. Annual reports on this registry have since been published in Japanese and publicized through the JBCS web site to active members of the JBCS [4].

**Fig. 1:**
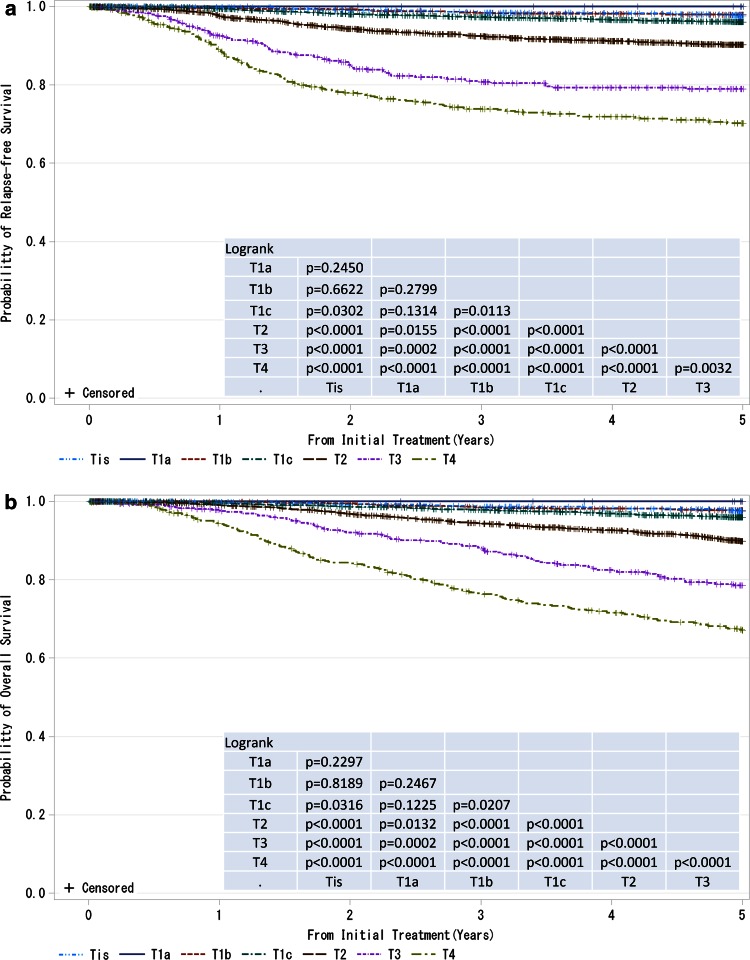
**a**, **b** Kaplan–Meier curves for relapse-free and overall survival of all cases by tumor classification (cT-category). *P* values were calculated using the log rank test. Tis: Non-invasive ductal carcinoma, lobular carcinoma in situ, or Paget disease; T1a: ≤0.5 cm; T1b: 0.5< tumor ≤1.0 cm; T1c: 1.0< tumor ≤2.0 cm, T2: 2.0< tumor ≤5.0 cm; T3: >5.0 cm; T4: tumor of any size with direct extension to the chest wall and/or skin (ulceration or skin nodules) or inflammatory carcinoma

**Fig. 2:**
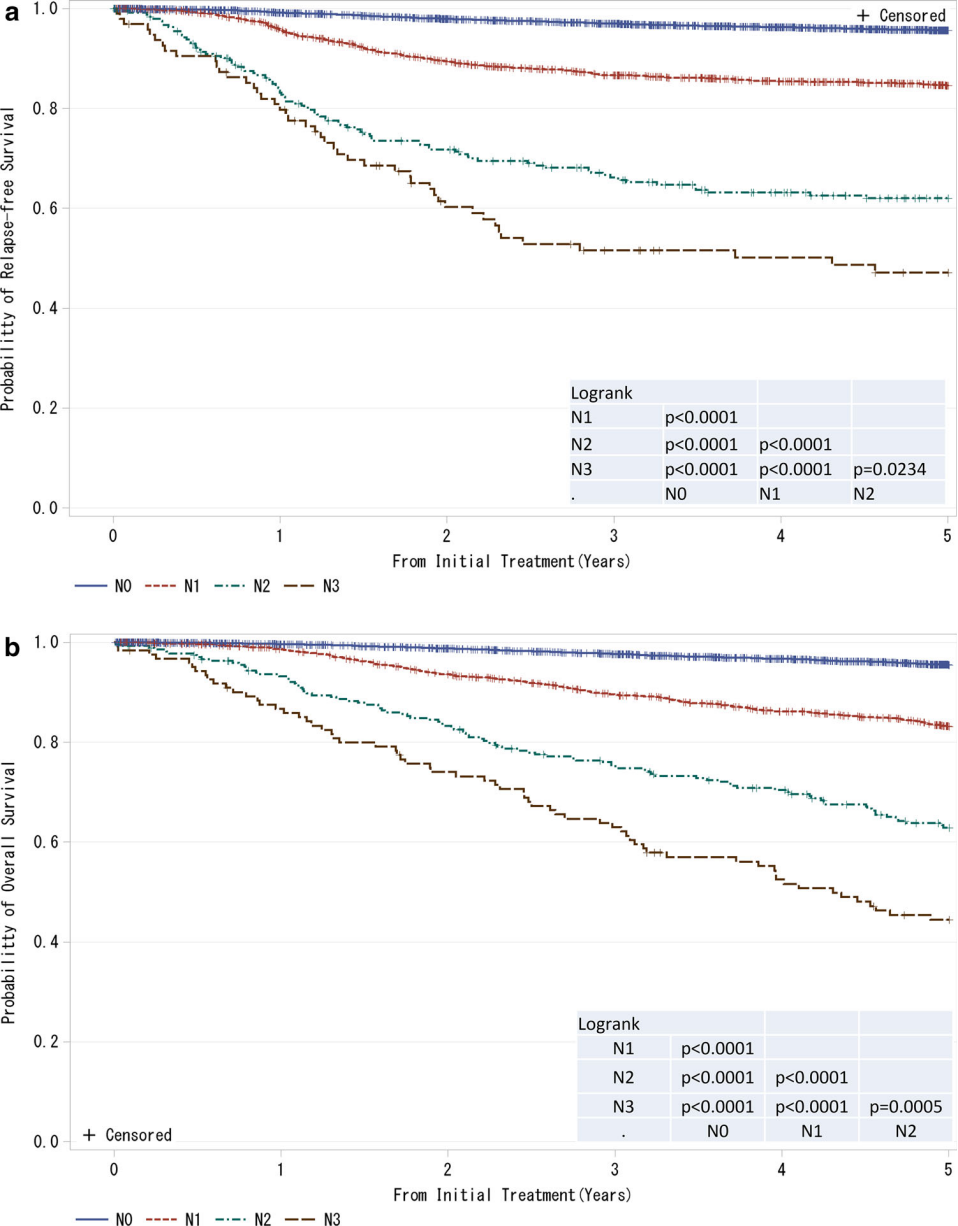
**a**, **b** Kaplan–Meier curves for relapse-free and overall survival of all cases by regional lymph nodes status (cN-category). N0: no regional lymph node metastases; N1: metastases in movable ipsilateral level I, II axillary lymph node(s); N2: metastases in ipsilateral level I, II axillary lymph nodes that are clinically fixed or matted OR metastases in clinically detected ipsilateral internal mammary nodes in the absence of clinically evident axillary lymph node metastases; N3: metastases in ipsilateral infraclavicular (level III axillary) lymph node(s) with or without level I, II axillary lymph node involvement OR metastases in clinically detected ipsilateral internal mammary lymph node(s) with clinically evident level I, II axillary lymph node metastases OR metastases in ipsilateral supraclavicular lymph node(s) with or without axillary or internal mammary lymph node involvement. *P* values were calculated using the log rank test

**Fig. 3:**
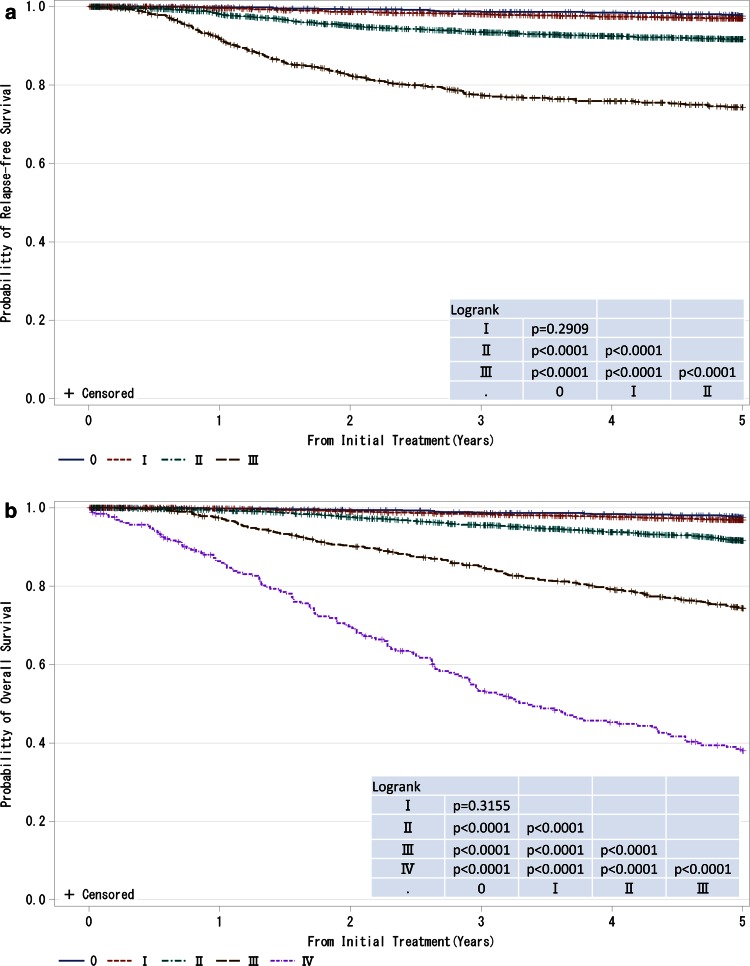
**a**, **b** Kaplan–Meier curves for relapse-free and overall survival of all cases by clinical stage (UICC). *P* values were calculated using the log rank test

**Fig. 4:**
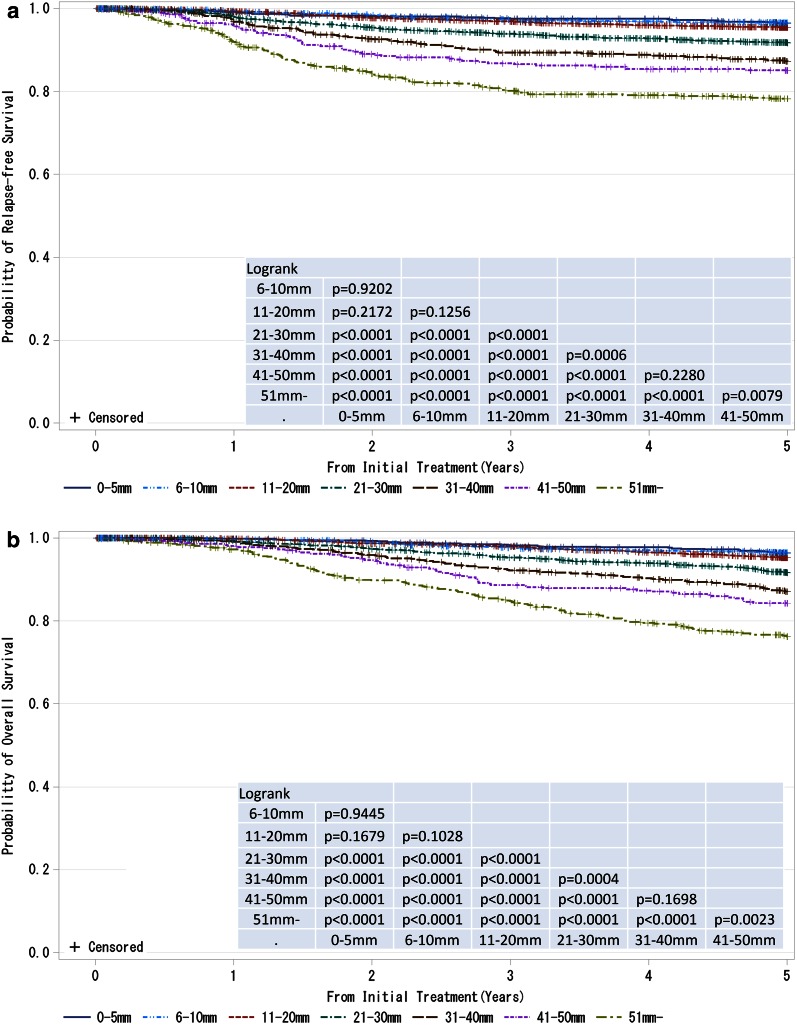
**a**, **b** Kaplan–Meier curves for relapse-free and overall survival of cases without neoadjuvant therapy by pathological tumor size (pT size). Tumor size is a marker of invasiveness. *P* values were calculated using the log rank test

**Fig. 5:**
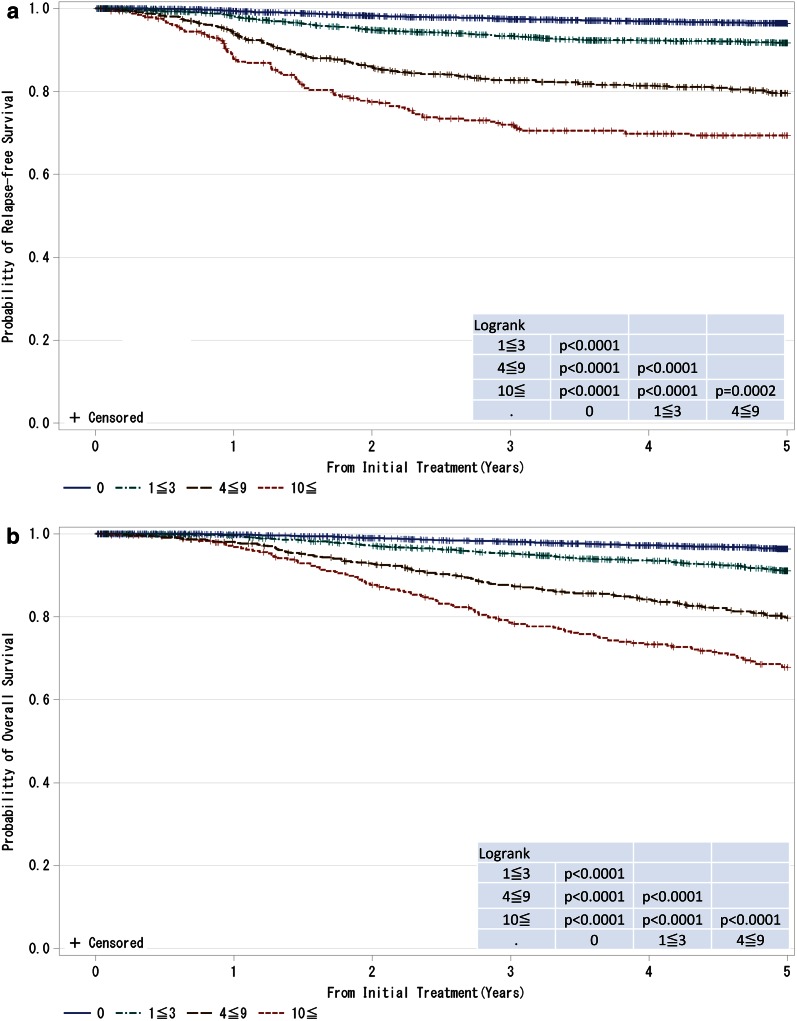
**a**, **b** Kaplan–Meier curves for relapse-free and overall survival of cases without neoadjuvant therapy by the number of metastatic lymph nodes. *P* values were calculated using the log rank test

**Fig. 6:**
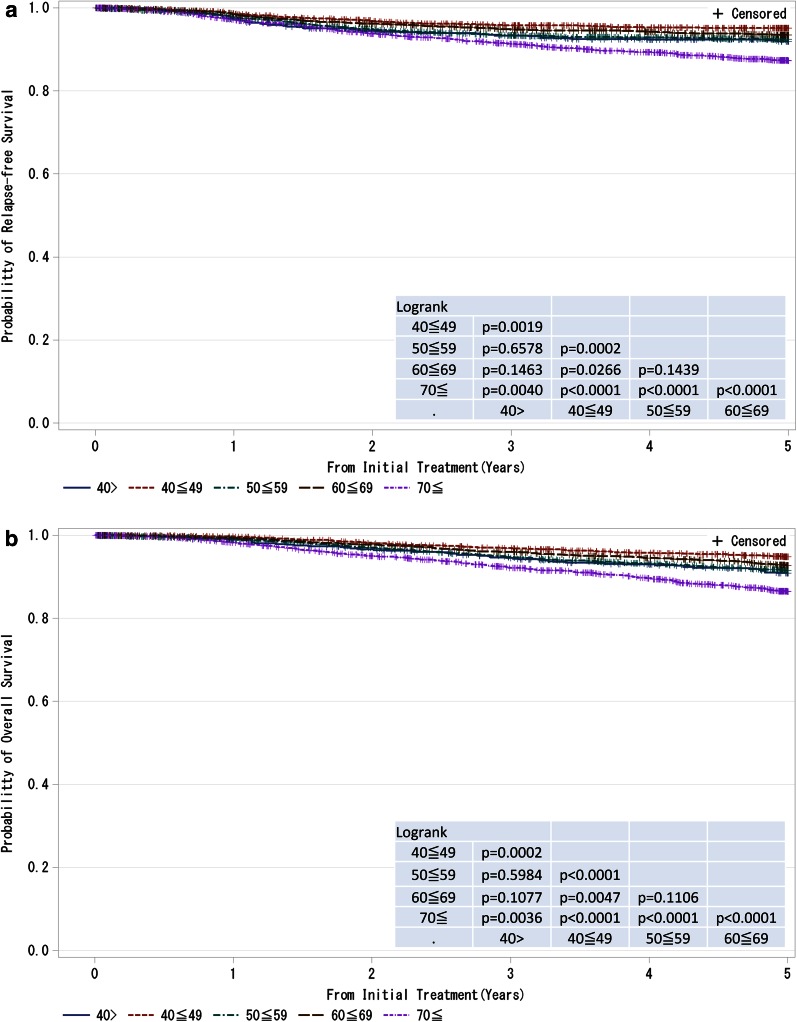
**a**, **b** Kaplan–Meier curves for relapse-free and overall survival of all cases by age. *P* values were calculated using the log rank test

**Fig. 7:**
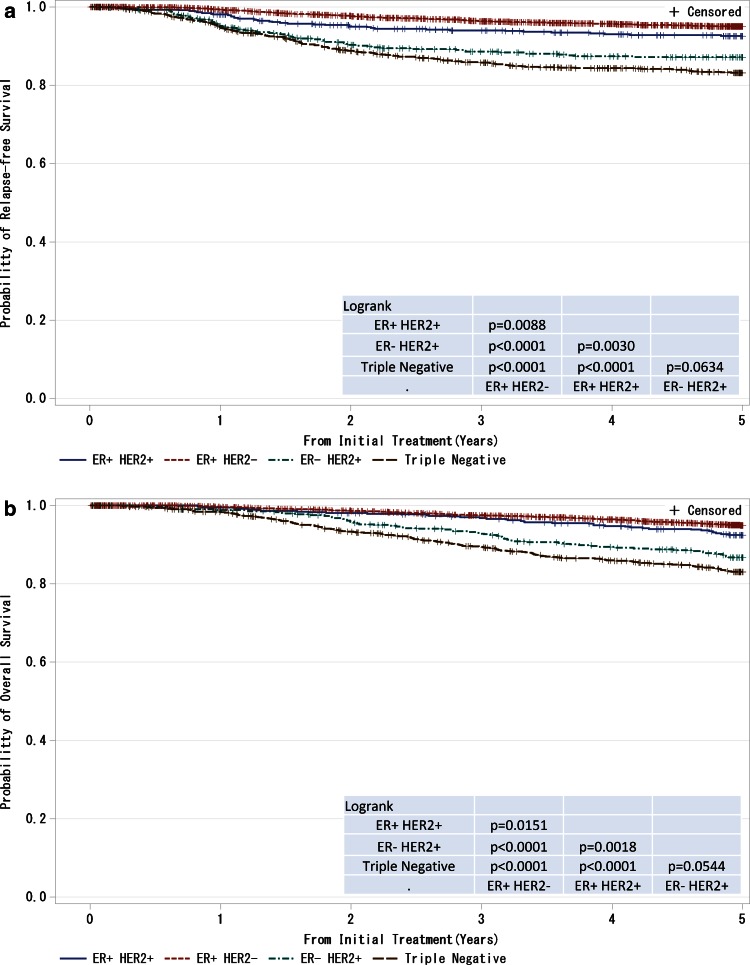
**a**, **b** Kaplan–Meier curves for relapse-free and overall survival of T1–T4, any N and M0 cases with respect to estrogen receptor (ER) status and HER2 (human EGFR-related 2) amplification status. *P* values were calculated using the log rank test. Relapse-free survival and overall survival of patients with respect to combined ER and HER2 status

**Fig. 8:**
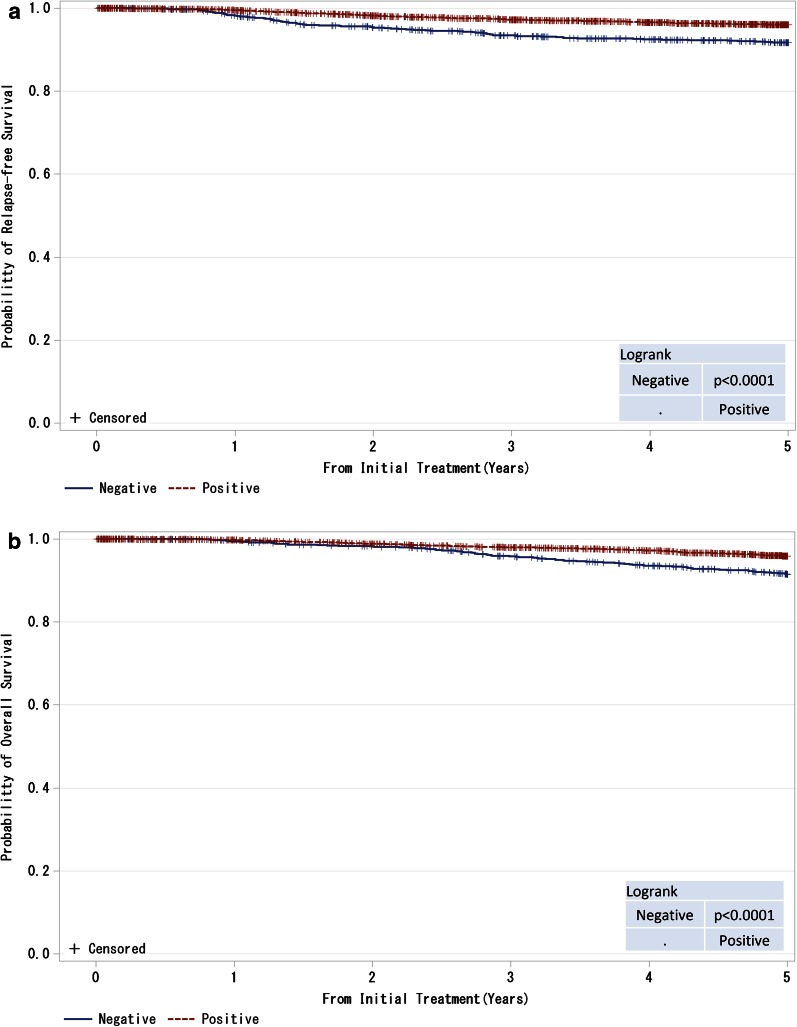
**a**, **b** Kaplan–Meier curves for relapse-free and overall survival of ER-positive and M0 cases by progesterone receptor (PgR) status. *P* values were calculated using the log rank test

**Fig. 9:**
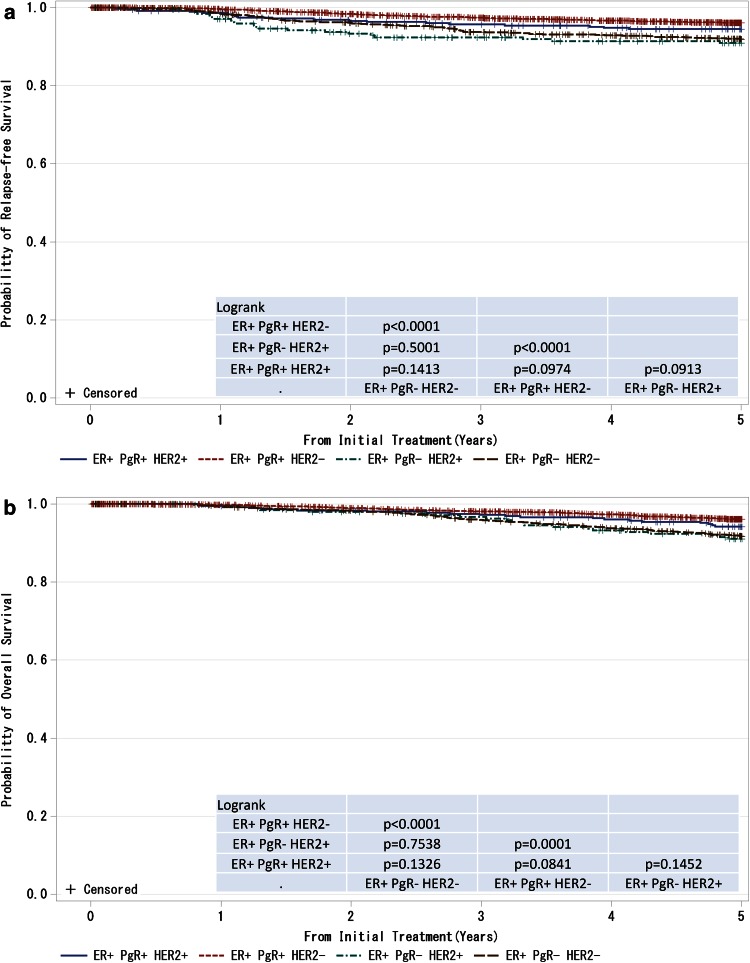
**a**, **b** Kaplan–Meier curves for relapse-free and overall survival of ER-positive and M0 cases with respect to PgR and HER2 amplifications. *P* values were calculated using the log rank test

We herein report the results of a 5-year prognostic analysis of cases registered in 2005 (Figs. 1, 2, 3, 4, 5, 6, 7, 8 and 9; Supplementary Tables 1–9). The number of facilities involved in the 2005 registration was 354 and the total number of cases was 20,786. The estimated incidence of breast cancer was reported to be 50,695 cases in 2005 by the National Cancer Center [5]. Therefore, approximately 41 % of newly diagnosed breast cancer patients were included in the JBCS registry in 2005. In this prognostic study, we analyzed 9971 cases in which the survival data were available from 161 facilities. The background characteristics of the patients are summarized in Table 1. The median follow-up period was 60.0 months (range 0.1–60.0 months). Note that during the study period, not only the cutoff levels of estrogen receptor and progesterone receptor but also their corresponding test procedures were non-standardized and that trastuzumab was rarely used because it was not covered by the Japanese National Health Insurance program as an adjuvant therapy for human epidermal growth factor receptor 2-positive breast cancer. On the whole, the survival data seem to be better than expected. However, it would be prudent to avoid commenting on any specific subject because the present study is part of an annual survival report. A data set spanning multiple years would be more suitable for addressing specific subjects, such as triple-negative type or HER2 type. This is planned for the next phase studies.

**Table 1:** Patient characteristics

Age		
Mean	SD	
57.36	12.81	
Tumor size(cm)		
Mean	SD	
2.64	2.12	
Tumor size^a^	Count	(%)
T0	118	1.18
Tis	914	9.17
T1a	61	0.61
T1b	836	8.38
T1c	2923	29.32
T2	3346	33.56
T3	459	4.6
T4	550	5.52
Unknown	764	7.66
N^a^		
N0	7616	76.38
N1	1791	17.96
N2	270	2.71
N3	121	1.21
Unknown	173	1.74
M^a^		
M0	9425	94.52
M1	257	2.58
Unknown	289	2.9
Stage^a^		
0	841	8.43
I	3386	33.96
II	3635	36.46
III	752	7.54
IV	257	2.58
Unknown	1100	11.03
ER		
Positive	7245	72.66
Negative	2325	23.32
Unknown	401	4.02
PgR		
Positive	5945	59.62
Negative	3594	36.04
Unknown	432	4.33
HER2		
Positive	1407	14.11
Negative	7351	73.72
Unknown	1213	12.17

*ER* estrogen receptor, *PgR* progesterone receptor, *HER2* human epidermal growth factor receptor 2

^a^ The TNM classification was identified by the UICC staging system

Cancer control activities vary among nations and regional areas due to differences in population structures. The life expectancy for Japanese women, 87 years old in 2012, has been the longest worldwide for several years [6]. In addition, Japan is a super-aging society, with 25 % of citizens being 65 years of age or above as of October 2013, which is the highest number among all other nations [7]. We believe that the outcomes of our registry provide significant information for countries that are expected to have a similar population structure to that of Japan in the near future.

## Electronic supplementary material

Supplementary material 1 (XLSX 21 kb)
